# Evaluation of the Response to Pulpal Sensibility Tests (Cold, EPT) in Anemic and Healthy Women

**DOI:** 10.1155/2022/3518817

**Published:** 2022-06-18

**Authors:** Maryam Kazemipoor, Mohammad Kazem Vatanchian, Sara Jambarsang, Fatemeh Owlia

**Affiliations:** ^1^Department of Endodontics, School of Dentistry, Shahid Sadoughi University of Medical Sciences, Yazd, Iran; ^2^Private Practice, Yazd, Iran; ^3^Department of Bio-Statistics and Epidemiology, Shahid Sadoughi University of Medical Science, Yazd, Iran; ^4^Department of Oral and Maxillofacial Medicine, School of Dentistry, Shahid Sadoughi University of Medical Sciences, Yazd, Iran

## Abstract

**Introduction:**

The prevalence of anemia has been reported high in the female population in Iran. Anemia can be asymptomatic or can present in a variety of symptoms, especially when serum Hb values decrease. The present study would assess dental pulp response to cold and EPT sensibility tests in anemic and healthy women.

**Materials and Methods:**

One hundred twenty maxillary central incisors belonging to participants aged 18–58 years were included in this survey. 60 patients had anemia (Hb ≤ 12.5) with/without medication (30 in each group) and 60 women as controls had no anemia (Hb > 12.5) with/without medication (30 in each group). Electric and cold pulpal sensibility tests were performed for all teeth. Statistical analysis was performed with t-student, Chi-square test, and two-way ANOVA. The significance level was set as *p* < 0.05.

**Results:**

According to the results of this study, the mean value of hemoglobin in anemic and healthy women was 11.5 and 14.08 g/dl, respectively. The mean value of response to EPT in anemic women with and without medication was 3.21 and 3.14, respectively. The mean value of response to EPT in healthy women with and without medication was 3.81 and 3.58 g/dl, respectively. The mean value of time delay response to cold test was 3.03 and 2.82 s in anemic patients with/without medication. Also, the mean value of time delay response to cold test was 2.80 and 2.93 s in healthy women with/without medication. The pulpal responses to EPT tests had significant differences between anemic and healthy women (*p*=0.043). There were no significant differences between anemic and healthy women considering time delay response to the cold test (*p*=0.077).

**Conclusion:**

Based on the results of the present study, tooth response to the EPT  sensibility test may alter in anemic patients despite medication. The evidence from this preliminary study suggests that peripheral neuropathy in anemic patients could affect pulpal sensibility tests.

## 1. Introduction

Iron deficiency anemia (IDA) is affecting more than a quarter of the world's population [1]. IDA is considered the most common nutritional deficiency anemia affecting approximately 30% of the general population and more than 50% of women of reproductive age [2]. Based on the definition of the World Health Organization, hemoglobin level less than 13 g/dL (130 g/L) in men and less than 12 g/dL (120 g/L) in women is considered anemia [3]. IDA can be asymptomatic or symptomatic, especially when serum Hb values decrease to 8–9.5 g/dL [4].

Iron as an essential element plays an important role in the metabolism of neurotransmitters and myelin formation, and its deficiency leads to dysfunction of peripheral nerve endings [5].

Iron influences nerve impulse conduction, and protein and enzyme synthesis which are essential for the synthesis of neurotransmitters including serotonin, norepinephrine, and dopamine [6].

Pulpal sensibility tests (thermal and electrical) are subjective and patient-related which indirectly assess the health status of dental pulp nerve fibers [7, 8]. Electrical pulp tester (EPT) creates ionic change in the fluid inside the dentinal tubules, which causes local depolarization and thus produces the action potential in healthy and intact delta A nerves fibers in the pulp-dentin complex [9]. Thermal sensibility tests cause tubular fluid movement, which stimulates the mechanoreceptors in the pulp-dentin complex [7].

EPT is particularly effective in cases with limited fluid flow through the dentinal tubules as a consequence of aging, dentine sclerosis, and calcification of the pulp space [10]. In comparison to the cold test, EPT should not be considered the primary choice for the assessment of pulp status because the cold test provides a more accurate response and it is easier to perform and interpret [9, 11]. Moreover, it has been reported that EPT was found to be more reliable in detecting healthy pulp tissues compared with diseased ones [12].

Materials available for cold tests include dry ice (CO_2_), ice, and refrigerant sprays (such as tetrafluoroethene, butane, propane, isobutene, dichlorofluoromethane (DDM), and ethyl chloride [12]. To detect a healthy pulp tissue, different cold tests have the same ability, but in diseased pulp because of the large differences in temperature reduction induced by CO_2_ snow and Endo-Ice, results were more diagnostically superior to ethyl chloride and ice sticks [12, 13]. Research has shown that the accuracy of CO_2_ and refrigerants to determine the sensibility of the pulpal nerve fibers is superior to the electrical test pulp [13].

Iron deficiency anemia caused insufficient oxygen transport by hemoglobin to peripheral tissues [14]. Mammalian neurons are sensitive to the availability of oxygen and transient reduction of oxygen triggers complex electrophysiological, hemodynamic, and biochemical cascades [14]. Hypoxia modified synaptic interaction and disturbed hemostasis characterized by enhanced cellular *K*^+^ efflux and Na^+^ and Ca^2+^ influx, followed by extracellular acidosis [15, 16]. Depolarization occurs shortly after hypoxia and induces neural hyperexcitability [14–18]. A higher concentration of Ca^2+^ in the cytosol may overstimulate Ca^2+^ dependent protease, phospholipase, and endonuclease [15]. Hypoxia also induced the release of transmitters and changes in membrane potential which leads to a reversible loss of neuronal function [14].

Normal functioning of the nervous system also depends on the barrier effect of the neural vasculature which is mainly attributed to the presence of complex tight junctions between endothelial cells [15]. Claudine 5, a key molecule in the tight junction assembly, was closely correlated with the increase in the trans endothelial electrical resistance. Hypoxia altered the location of the protein in the plasma membrane which results in a decrease in the trans endothelial electrical resistance [15].

Nerve endings in the dental pulp tissue can be affected by neurological changes occurred in anemia due to other peripheral sensory nerves, and therefore, the response of pulpal nerves to sensibility tests could be altered in anemic patients. The aim of the present study was to investigate the response of dental pulp nerve fibers to pulpal sensibility tests (cold and electrical) in anemic and healthy women.

## 2. Materials and Methods

**Figure 1:**
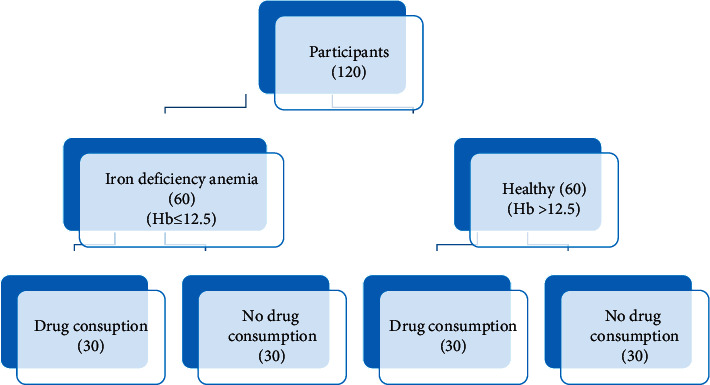
Details of participants in the study in different subgroups.

All the experimental procedures in the present study were approved by the Ethics Committee of Research Shahid Sadoughi University of Medical Sciences, Yazd (IR.SSU.REC.1399.118). Informed consent was obtained from each patient before initiating the study. One hundred twenty women (*n* = 120) aged between 18 and 54 years have participated in this descriptive cross-sectional study (Figure 1). The patients included in this study had been admitted to the specialized clinic in Yazd. Patients with a history of any systemic diseases or medication other than iron deficiency anemia, taking tricyclic antidepressants, anticonvulsants, and antihypertensive medication during the last 3 months, the use of different amounts and types of analgesics 48 hours before the sensibility tests were excluded from the present study. The two groups were matched according to their age. Local factors composed of extensive filling, dental caries, history of trauma or orthodontic treatment, and periodontal problems were also considered. Sensibility tests were applied to the maxillary intact central teeth without any restoration, caries lesion, periodontal problem, sensitivity to percussion, history of trauma, and orthodontic treatment. Before testing, the surface of the teeth was made free of debris, calculus, and plaque. Teeth were first dried and isolated with a cotton roll, and an electrocardiography gel (BP Ultra Gel, Turkuaz Saglik co, Turkey) was applied on the buccal face of the crown as an interface media. An electric pulp tester (EPT) (Gentle-pulse, Parkell, USA) probe was placed on the sound coronal third of the labial surface and the “tingling” sensation felt by the patient once the increasing voltage reaches the pain threshold was recorded. Cold testing with ethyl chloride was accomplished by using a large cotton pellet on the buccal surface of the tooth for 15 seconds, or until patient indicated a response. The time interval between the cold application and patient response was recorded.

### 2.1. Statistical Analysis

Data were analyzed by the statistical software IBM SPSS *v*. 22. Results were reported with descriptive indices as mean and standard deviation. Inferential statistics were applied with the use of Student's t-test, two-way ANOVA model, and regression model. The significant level was set as *p* < 0.05.

## 3. Results

In this descriptive cross-sectional study, 120 female patients with a mean age of 34.45 ± 10.71 and age range of 18–54 years who were referred to a specialized clinic have participated (Table 1). There was no significant difference between groups considering the age factor (*p* value = 0.349).

According to the study design, 60 women without iron deficiency anemia (Hb > 12.5) with and without drug consumption (*n* = 30) and 60 women with iron deficiency anemia (Hb ≤ 12.5) with and without drug consumption (*n* = 30) have participated in the present research. The mean hemoglobin level in the anemic group was 11.50 g/dl with a range of changes from 8.91 to 12.42 (Table 1). In the nonanemic group, the mean hemoglobin level was 14.08 g/dl with a range of changes from 12.52 to 17.43 (Table 1).

The mean ± SD of scores in responses to the EPT test and the time delay response to the cold test in the experimental groups are presented in Table 1. According to the results of pulse oximetry in the anemic and healthy women, there was no significant difference between the two groups (*p* value = 0.093).

A two-way analysis of variance was used to investigate the relationship between anemia and response time to cold test and electrical pulp tester. In this analysis, iron supplementation was used as a moderator. Based on the two-way ANOVA test, the pulp tester mean score in the anemia group was significantly lower (*p*. value = 0.043). This result indicates that even despite treatment with iron tablets in the anemic group, the response to the electrical pulp tester is impaired. There was no significant difference between the two groups considering the time delay response to the cold test (*p* value = 0.077).

## 4. Discussion

Iron deficiency anemia is the most common hematological disorder and is a consequence of chronic blood loss or lack of iron consumption in diet [19]. Approximately 50% of anemia cases are considered to be due to iron deficiency, but the prevalence of this disease varies based on population group and different areas [20]. The prevalence of iron deficiency anemia has been reported high in the female population in Iran (52.3%) [21]. Also, the prevalence of this disease is much higher in women compared with men and is considered a contributing factor for the higher incidence of postendodontic pain in women [20, 22]. Up to the present, there is no study on the relationship between iron deficiency anemia and the response to pulpal sensibility tests.

Based on the results of the present study, anemic women in comparison to healthy ones showed a lower threshold in response to the EPT test. Anemic patients with medication also revealed a higher threshold in response to EPT compared with anemic participants without medication. Considering time delay in response to the cold test, anemic patients responded with more delay. Anemic patients with medication also had more delays in response to the cold test.

Hypoxia and the effect of this phenomenon on the neurophysiology and responsiveness of dental pulp nerve fibers have been studied in a limited study [23–27]. It has been shown that, in sickle cell anemia, hypoxia causes systemic complications such as local vascular contraction and periods of pain that can cause damage to vital organs [25]. In the oral cavity, the disease can cause ischemia and necrosis of the facial bones, and osteomyelitis without dental origin, especially in the mandible [25, 27]. It has also been shown that blockage of dental pulp microvasculature in healthy teeth may cause pulpal inflammation [25] and even pulpal necrosis [23, 27].

In the absence of systemic diseases, alteration in tissue pressure following a decrease in pulpal blood flow and hypoxia in the inflamed pulp can affect the nerve-free endings, resulting in damage to and stimulation of the pulpal nerves [24, 26].

Iron deficiency anemia caused insufficient oxygen transport by hemoglobin to peripheral tissues [28]. Mammalian neurons are sensitive to the availability of oxygen, and transient reduction of oxygen triggers complex electrophysiological, hemodynamic, and biochemical cascades [14]. Hypoxia modified synaptic interaction and disturbed on hemostasis characterized by enhanced cellular *K*^+^ efflux and Na^+^ and Ca^2+^ influx followed by extracellular acidosis [18]. Depolarization occurs shortly after hypoxia and induces neural hyperexcitability [14]. A higher concentration of Ca^2+^ in the cytosol may overstimulate Ca^2+^-dependent protease, phospholipase, and endonuclease [16]. Hypoxia also induced the release of transmitters and changes in membrane potential which leads to a reversible loss of neuronal function [14]. Normal functioning of the nervous system also depends on the barrier effect of the neural vasculature which is mainly attributed to the presence of complex tight junctions between endothelial cells [15]. Claudine 5, a key molecule in the tight junction assembly, was closely correlated with the increase in the trans endothelial electrical resistance. Hypoxia altered the location of the protein in the plasma membrane which results in a decrease in the trans endothelial electrical resistance [14].

The aforementioned mechanisms might explain the alteration in the response of anemic sensory nerves to pulpal sensibility tests. In the present study, the comparison of the response of dental pulp nerve fibers to pulpal sensibility tests (cold and electrical) in anemic and healthy women was investigated. There was no significant difference between the two groups considering the time delay in response to the cold test. On the contrary, the response to EPT was disturbed in anemic women, even with medication. As mentioned before, hypoxia decreased the trans endothelial electrical resistance and increased the excitability of nerve fibers. The lower response threshold in response to the EPT test which was observed in anemic patients may be a consequence of tissue hypoxia in anemia. It has been shown that even with medication, the response to the EPT test is disturbed in anemic patients.

The results of pulse oximetry in the present study have also shown that there was no significant difference between anemic and healthy women, although the level of saturation was higher in healthy women. Fluctuation in the blood oxygen saturation and the level of impact on nerve excitability should be assessed in future studies.

The mechanism of evoking in the two pulpal sensibility tests is different. Thermal sensibility tests create the movement of intratubular fluid based on hydrodynamic, but the electrical pulp tester directly stimulates the nerve endings [29]. The lower response threshold to the EPT test that has been observed in anemic women may be attributed to the fluctuation of sexual hormones, nutritional deficiency, and other molecular changes in extracellular substances and neural cell membranes as the consequences of anemia.

There were significant differences between groups in the response to EPT. However, the mean values recorded for the EPT test may be statistically significant between anemic and nonanemic women, but it seems that the decimal increase in EPT score is not of any clinical value.

The sample size of the present study was 120, more than some studies on the evaluation of sensibility tests in systemic diseases [30, 31] and similar to Tavakolinejad [32]. Our strict inclusion criteria were one of the priorities of this study toward others. Since the health status could impact the test results, patients with systemic diseases other than anemia did not participate as a study group in the present study.

Patients were not entered into the evaluation process if they had a history of taking tricyclic antidepressants, anticonvulsants, and antihypertensive medication during the last 3 months [33]. Systemic doses of different types of analgesics during 48 hours can also alter the EPT responses. Some of the local factors that could impact on the pulp responses composed of extensive filling, dental caries, history of trauma, and periodontal problems. Periodontal problems, less enamel thickness, carious teeth, and a history of trauma could induce bias in the results of the EPT test [34]. In the present study, sound anterior teeth without a history of trauma were selected for sensibility tests.

EPT, as one of the reliable and accurate tests for assessment of healthy A*ð* nerve fibers in the pulpal complex, and the cold test, as the most repeatable sensibility test, are diagnostic tools for the evaluation of pulpal disorders [34]. EPT is a useful method particularly in teeth with limited fluid flow through dentinal tubules, such as with dentine sclerosis [35]. Aging has a negative impact on the results of EPT and cold tests [35]; therefore, the mean age of participants in both groups was similar with no significant differences. According to the multiple linear regression analysis, withholding other variables on response time to cold test only aging had a significant positive relationship. It means that the sensory response threshold to cold increases with aging. It could be explained by the deposit of secondary dentin and limited fluid movement in dentinal tubules of aged dentin [34].

Due to more accessibility, easier isolation, lower caries, and point connection to adjacent teeth, anterior teeth were more suitable for the present study. In this study, the central maxillary incisor was selected because central teeth have a lower threshold for EPT rather than other anterior teeth [36]. Direct pathway of the dentinal tube, more concentration of neural components, low enamel thickness, and low voltage required to stimulus led to the selection of one-third incisal edge of teeth for vitality pulp tests [10, 37].

There were significant differences between healthy and anemic women considering RDW (Red Cell Distribution Width) in the present study. It has been shown that in the latent stage of iron deficiency, the RDW index was significantly higher in comparison to MCV, MCH, and MCHC indices. RDW had sensitivity 82.3% and specificity 97.4% [38],whereas MCV, MCH, and MCHC had 29.2%, 68.1%, and 15% sensitivity, but specificity was 98.7%, 83.1%, and 96.1% in the detection of iron deficiency [38]. Iron deficiency anemia without other complicating diseases could be screened out early by increased RDW when RBC indices were normal. The higher values of RDW in anemic women in the present study showed the purity of data and could rule out the presence of other complications such as minor thalassemia.

It seems that changes in EPT responses could be interpreted as a change in pulpal nerve conduction. It should be analyzed besides the results of other thermal tests and it does not show any histologic data about pulp conditions [39].

Sensibility tests applied in the present study are subjective and recorded by self-declaration of the patients. Since the psychogenic part of pain perception and response to stimulus injuries plays an important role in the results of subjective tests, it is suggested to use objective vitality tests such as laser Doppler flowmetry and pulse oximetry for better assessment of the changes in the pulpal neural network. Also, the characteristic of the study population in regard to other blood factors and other types of anemia should be evaluated for exact analysis of the effect of anemia on the response of pulpal nerve fibers. Despite the limitations, the study is important and novel as a first step for designing future experiments.

## 5. Conclusions

According to the results, tooth responses to the EPT sensibility test were disturbed in anemic women. Anemic patients showed a lower sensory response threshold to EPT than in the healthy group.

## Figures and Tables

**Table 1:** Comparison of the response to pulpal sensibility tests between anemic and healthy women.

	Anemic women		Healthy women	*p* value^*∗*^	Adjusted *p* value^*∗∗*^	Adjusted *pp* value^†^	Healthy women
	Mean	SD	Mean	SD			
Age	35.47	10.649	33.63	10.734	0.349	—	—
Hemoglobin	11.50	0.879	14.08	1.06	<0.001	<0.001	<0.001
Pulp tester	3.17	1.36	3.69	1.41	0.043	<0.001	<0.001
Time delay to cold	2.93	1.06	2.87	1.17	0.769	0.770	0.949
Pulse oximetry	0.96	0.01	0.97	0.01	0.093	0.094	0.125

^*∗*^Comparison of the two groups after adjusted on anemia treatment, using *t*-test. ^*∗∗*^Comparison of the two groups after adjusted on anemia treatment, using regression. ^†^Comparison of the two groups after adjusted on anemia treatment and age, using regression.

## Data Availability

The datasets used and/or analyzed during the current study are available from the corresponding author on reasonable request.
